# Associations Between Patient-Reported Nutritional Status, Toxicity, and Survival in Limited-Stage SCLC

**DOI:** 10.1016/j.jtocrr.2024.100764

**Published:** 2024-11-12

**Authors:** Evgenia Taranova, Marianne Aanerud, Tarje O. Halvorsen, Kristin T. Killingberg, Marit Slaaen, Bjørn H. Grønberg

**Affiliations:** a Department of Clinical Science, University of Bergen, Bergen, Norway; b Department of Thoracic Medicine, Haukeland University Hospital, Bergen, Norway; c Department of Clinical and Molecular Medicine, NTNU, Norwegian University of Science and Technology, Trondheim, Norway; d Department of Oncology, St. Olavs Hospital, Trondheim, Norway; e The Research Centre for Age-Related Functional Decline and Disease, Innlandet Hospital Trust, Ottestad, Norway; f Faculty of Medicine, Institute of Clinical Medicine, University of Oslo, Oslo, Norway

**Keywords:** Small-cell lung cancer, Chemoradiotherapy, Weight-loss, PG-SGA SF, Nutrition

## Abstract

**Introduction:**

In general, malnutrition is associated with more treatment toxicity and shorter survival in patients with cancer, but little is known about its impact on limited-stage (LS) SCLC. We investigated whether nutritional status and weight loss were associated with treatment outcomes in a randomized trial of thoracic radiotherapy (TRT) in LS SCLC (NCT02041845, N = 170).

**Methods:**

Patients received platinum-etoposide-chemotherapy and were randomized to receive TRT of 60 Gy in 40 fractions or 45 Gy in 30 fractions. They reported nutritional status on the Patient-Generated Subjective Global Assessment Short Form (PG-SGA SF) and were categorized as having low (PG-SGA SF score 0–3), intermediate (score 4–8), or high (score ≥ 9) malnutrition risk.

**Results:**

In total, 113 patients who completed the PG-SGA SF at baseline and received one or more fractions of TRT were analyzed. Median PG-SGA SF score was 3.0; 52.2% had low, 29.2% intermediate, and 18.6% had high malnutrition risk; and 22.1% had 5% or more weight loss three months before enrolment. There were no significant differences in grade 3 to 4 toxicity (low: 88.1%, intermediate: 90.9%, high: 85.7%; *p* = 0.86), median progression-free survival (low: 15.8 months, intermediate: 11.8 months, high: 47.0 months; *p* = 0.25) or median OS (low: 35.5 months, intermediate: 26.8 months, high: 47.0 months; *p* = 0.24) across malnutrition categories. Weight loss was not significantly associated with grade 3 to 4 toxicity (≥5%: 92.0%, <5%: 87.0%; *p* = 0.73), median progression-free survival (≥5%: 24.0 months, <5%: 15.9 months; *p* = 0.51) or median OS (≥5%: 30.6 months, <5%: 35.5 months; *p* = 0.74).

**Conclusion:**

Patient-reported nutritional status and weight loss before concurrent chemoradiotherapy were neither associated with toxicity nor survival.

## Introduction

SCLC accounts for 13% to 15% of all lung cancer cases.^1^ Overall, the prognosis is poor, but up to 40% of patients with limited-stage (LS) disease are cured by concurrent platinum-based chemotherapy and thoracic radiotherapy (TRT) followed by prophylactic cranial irradiation (PCI) to responders.^2^ Nevertheless, population-based studies show that not all patients with LS SCLC receive concurrent chemoradiotherapy (CRT),^3^^,^^4^ and although twice-daily TRT is the most recommended schedule,^2^^,^^5^^,^^6^ only a minority of patients actually receive two fractions per day.^7^ The main reasons for offering other TRT schedules are probably concerns about severe radiotoxicity, especially esophagitis,^3^ and logistical challenges.^5^^,^^8^

Weight loss and poor nutritional status are common among patients with cancer and are associated with more treatment toxicity, poor treatment response, and shorter survival.^9^, ^10^, ^11^, ^12^ As TRT might cause esophagitis and dysphagia,^9^ which often hinders food intake,^13^^,^^14^ and because reduced appetite and nausea are common side-effects of chemotherapy, weight loss and poor nutritional status before the start of chemoradiotherapy are concerns when considering potentially curative chemoradiotherapy in locally advanced NSCLC.^15^ Nevertheless, few have investigated whether or to what extent this should be taken into consideration when considering patients with LS SCLC for CRT. In general, SCLC responds better and faster to CRT than most other patients with solid tumors (including NSCLC).^16^

The Patient-Generated Subjective Global Assessment Short Form (PG-SGA SF, Copyright FD Ottery, pt-global.org),^17^ is a validated tool for screening, assessing, and monitoring malnutrition in patients with cancer.^18^, ^19^, ^20^, ^21^, ^22^, ^23^ Higher scores on PG-SGA SF are associated with cancer cachexia, longer hospital stays, reduced quality of life, and shorter survival in patients with cancer, including NSCLC.^22^^,^^24^ Nevertheless, it is not known whether this is the case in LS SCLC.

We assessed pre-treatment weight loss and nutritional status using the PG-SGA SF among participants in our randomized phase II clinical trial comparing high-dose with standard-dose twice-daily TRT in LS SCLC,^25^ and analyzed whether weight loss and patient-reported nutritional status before treatment were associated with toxicity, progression-free survival (PFS), and overall survival (OS).

## Methods and Materials

### Ethical Approvals

The trial was approved by the Regional Committee for Medical Research Ethics, Central Norway, the National Committee on Health Research Ethics in Denmark, and the Regional Ethics Board in Gothenburg, Sweden (ClinicalTrials.gov: NCT02041845). All patients provided written informed consent.

### Patients, Treatment, and Assessment

A description of the design of our trial, eligibility criteria, and study treatment were published earlier.^25^ Briefly, patients aged 18 years and older with performance status (PS) 0 to 2 and LS SCLC (negative cytology was required if pleural fluid was present) were to receive four courses of platinum-etoposide chemotherapy and were randomized to receive twice daily TRT of 60 Gy in 40 fractions or 45 Gy in 30 fractions. Responders to CRT were offered PCI of 25 to 30 Gy in 10 to 15 fractions. Patients in the 60 Gy group had significantly longer median OS (43.5 versus 22.5 months; *p* = 0.037).

Treatment response was assessed according to the Response Evaluation Criteria in Solid Tumors 1.1 within three weeks after completion of CRT. Toxicity was assessed according to the Common Terminology Criteria for Adverse Events version 4.0 on study visits throughout the treatment- and follow-up period. Patients reported weight loss during the last three months before enrolment (categorized as <5% or ≥5% of their body weight) and completed the PG-SGA SF questionnaire on paper in their native language (Norwegian, Danish, or Swedish) before treatment commenced.

Patients who completed the PG-SGA SF at baseline and received at least one fraction of TRT were included in the present study.

### The PG-SGA Short Form

The PG-SGA was developed for screening, assessing, and monitoring malnutrition in patients with catabolic conditions, including cancer.^17^^,^^23^^,^^26^ The first part of PG-SGA, the PG-SGA short form (SF), is completed by the patient only, and the second part is completed by healthcare personnel.

The PG-SGA SF (Supplementary Fig. 1) includes four components: weight history within specified intervals during the last six months (Box 1; 0 to 5 points); food intake the past month (Box 2; 0 to 4 points); nutrition impact symptoms that interfere with food intake, such as poor appetite, mouth sores, changes in taste and smell, feeling full quickly, nausea, constipation, and diarrhea (Box 3; 0 to 24 points); and physical activity and function (Box 4; 0 to 3 points). Total scores range from no problems (0 points) to major problems (36 points).

Patients were categorized as having low (PG-SGA SF score 0–3), intermediate (score 4–8), or high (score ≥ 9) risk of malnutrition similar to previous validation studies and PG-SGA SF triage recommendations.^17^^,^^19^^,^^27^, ^28^, ^29^

### Statistical Considerations

Kruskal-Wallis H test was used for testing differences in mean number of chemotherapy courses received, and Fisher’s exact test was used for comparisons of treatment completion and treatment toxicity across malnutrition risk and weight loss categories. Logistic regression was used to analyze if PG-SGA SF score, malnutrition risk category, and weight loss were associated with grade 3 to 4 toxicity.

Survival was estimated using the Kaplan-Meier method. OS was defined as the time from chemotherapy commenced until the death of any cause. Progression-free survival was defined as the time from chemotherapy commenced until disease progression or death of any cause. Follow-up was censored at the last observation alive (for OS) or last computed tomography evaluation (for PFS). Follow-up time for OS was estimated using the reverse Kaplan-Meier Method. Survival was compared for malnutrition risk categories (low, intermediate, and high) and weight loss (<5% or ≥5% the last three months before commencing treatment) using the log-rank test. Cox proportional hazard regression was used for univariable and multivariable PFS and OS analysis of PG-SGA SF score, malnutrition risk, and weight loss. Univariable analyses split by treatment arm were performed to explore the potential impact of the TRT schedule. All multivariable regression models were adjusted for baseline characteristics (sex, age, disease stage [I–II versus III], and PS) and treatment arm.

All analyses were two-sided, and the significance level was defined as a *p* less than 0.05. Statistical analyses were performed in RSstudio (Version 2022.12.0+3539, Vienna, Austria).

## Results

### Patients

**Figure 1:**
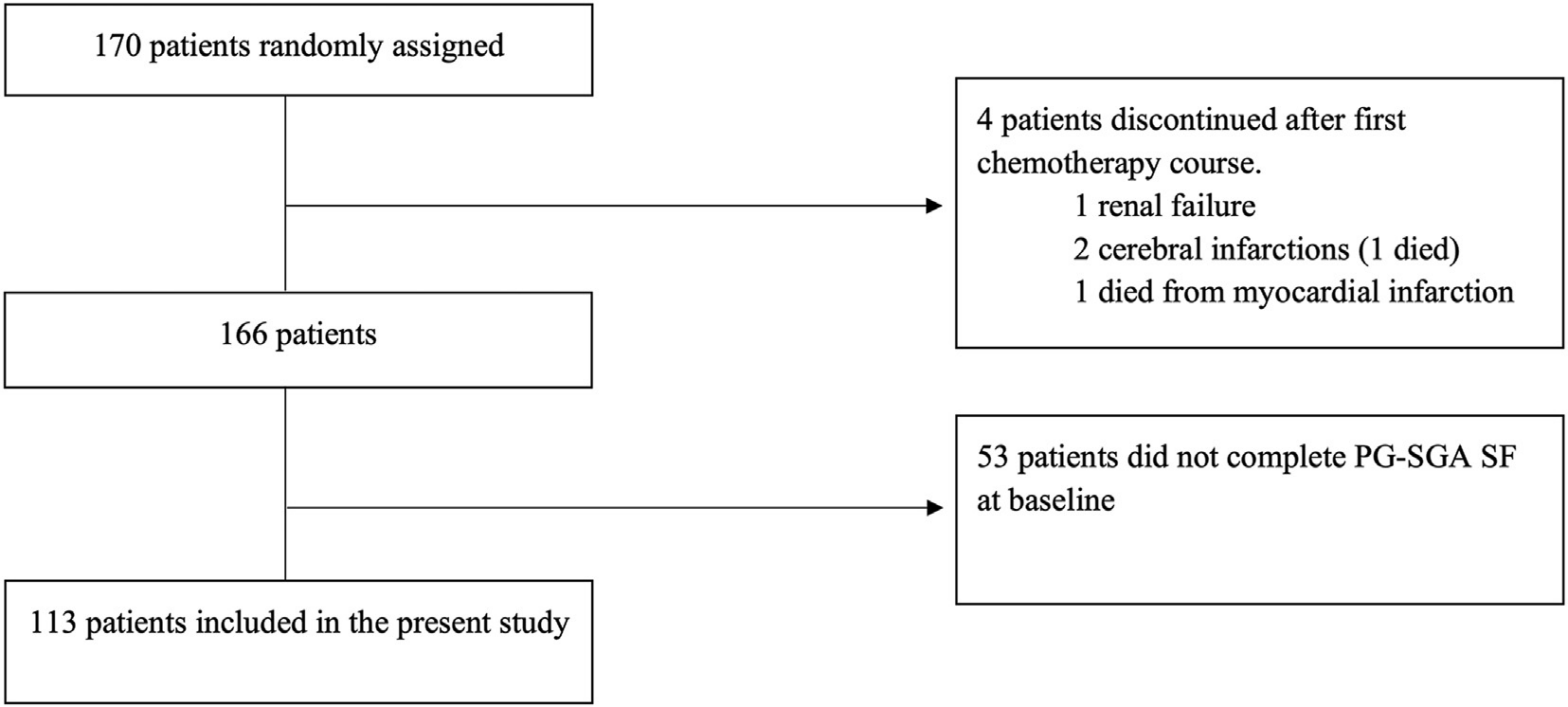
Patient selection. PG-SGA SF, Patient-Generated Subjective Global Assessment Short Form.

Between July 8, 2014, and June 6, 2018, 170 eligible patients were randomized. Of these, 113 patients (66.5%) completed the PG-SGA SF at baseline, received one or more TRT fractions, and were included in the present study (Fig. 1). Of these, 102 patients have available data on weight loss 5% or less or less than 5% three months pre-enrolment. The median follow-up time for OS was 90.2 months (interquartile range: 75.8–95.1).

Baseline characteristics and treatment completion for all patients according to malnutrition risk are presented in Table 1. Overall, the median age was 65 years (range: 48–80), 31.9% were 70 years or older, 54.0% were female individuals, 88.5% had Eastern Cooperative Oncology Group PS 0–1, 87.6% had stage III disease, 98.1% were current or former smokers and 9.7% had pleural effusion. Twenty-five patients (22.1%) experienced weight loss of 5% or higher in the last three months before enrolment, whereas 77 patients (68.1%) did not.

**Table 1:** Baseline Characteristics

Baseline Characteristics	Low Malnutrition Risk (n = 59)	Intermediate Malnutrition Risk (n = 33)	High Malnutrition Risk (n = 21)	Total Valid PG-SGA SF (n = 113)	Missing PG-SGA-SF (n = 53)
Age					
Mean	64.7 (7.08)	66.9 (7.27)	64.0 (8.37)	65.2 (7.41)	63.4 (9.30)
Median	65.0 [48.0–79.0]	68.0 [52.0–80.0]	63.0 [48.0–78.0]	65.0 [48.0–80.0]	64.0 [36.0–81.0]
>70 y	18 (30.5)	12 (36.4)	6 (28.6)	36 (31.9)	14 (26.4)
Sex					
Female	30 (50.8)	19 (57.6)	12 (57.1)	61 (54.0)	35 (66.0)
Male	29 (49.2)	14 (42.4)	9 (42.9)	52 (46.0)	18 (34.0)
Disease stage (TNM v.7)					
I and II	9 (15.3)	5 (15.2)	0 (0)	14 (12.4)	13 (24.5)
III	50 (84.7)	28 (84.8)	21 (100)	99 (87.6)	40 (75.5)
ECOG performance status					
0	31 (52.5)	12 (36.4)	5 (23.8)	48 (42.5)	30 (56.6)
1	24 (40.7)	17 (51.5)	11 (52.4)	52 (46.0)	20 (37.7)
2	4 (6.8)	4 (12.1)	5 (23.8)	13 (11.5)	3 (5.7)
Pleural fluid present					
Yes	4 (6.8)	2 (6.1)	5 (23.8)	11 (9.7)	2 (3.8)
No	54 (91.5)	31 (93.9)	16 (76.2)	101 (89.4)	51 (96.2)
Missing	1 (1.7)	0 (0)	0 (0)	1 (0.9)	0 (0)
Smoking history					
Current	38 (64.4)	22 (66.7)	17 (81.0)	77 (68.1)	34 (64.2)
Former	19 (32.2)	10 (30.3)	4 (19.0)	33 (29.2)	18 (34.0)
Never	1 (1.7)	1 (3.0)	0 (0)	2 (1.8)	1 (1.9)
Data missing	1 (1.7)	0 (0)	0 (0)	1 (0.9)	0 (0)
Median pack years for current or former smokers, range	32.0 [13.0–60.0]	30.0 [4.00–61.0]	30.0 [10.0–50.0]	30.0 [4.00–61.0]	40.0 [10.0–273]
Data missing	18 (30.5)	8 (24.2)	3 (14.3)	29 (25.7)	15 (28.3)
Treatment group					
45 Gy	29 (49.2)	16 (48.5)	8 (38.1)	53 (46.9)	24 (45.3)
60 Gy	30 (50.8)	17 (51.5)	13 (61.9)	60 (53.1)	29 (54.7)
Number of chemotherapy courses					
1	1 (1.7)	0 (0)	0 (0)	1 (0.9)	1 (1.9)
2	2 (3.4)	1 (3.0)	1 (4.8)	4 (3.5)	2 (3.8)
3	2 (3.4)	2 (6.1)	0 (0)	4 (3.5)	1 (1.9)
4	54 (91.5)	30 (90.9)	20 (95.2)	104 (92.0)	49 (92.5)
Mean	3.85 (0.551)	3.88 (0.415)	3.90 (0.436)	3.87 (0.491)	3.85 (0.568)
Completed thoracic radiotherapy as planned	57 (96.6)	32 (97.0)	20 (95.2)	109 (96.5)	51 (96.2)
Received PCI					
Yes	46 (78.0)	28 (84.8)	17 (81.0)	91 (80.5)	45 (84.9)
No	11 (18.6)	4 (12.1)	3 (14.3)	18 (15.9)	7 (13.2)
Data missing	2 (3.4)	1 (3.0)	1 (4.8)	4 (3.5)	1 (1.9)
Total numerical PG-SGA SF score	2.00 [0–3.00]	6.00 [4.00–8.00]	13.0 [9.00–25.00]	3.00 [0–25.00]	
Weight loss ≥ 5%					
Yes	6 (10.2)	7 (21.2)	12 (57.1)	25 (22.1)	8 (15.1)
No	48 (81.4)	22 (66.7)	7 (33.3)	77 (68.1)	32 (60.4)
Data missing	5 (8.5)	4 (12.1)	2 (9.5)	11 (9.7)	12 (24.5)

Data are mean (SD), median [min, max] or n (%).

ECOG, Eastern Cooperative Oncology Group; PCI, prophylactic cranial irradiation; PG-SGA SF, Patient-Generated Subjective Global Assessment Short Form; TNM, tumor, node, metastasis.

**Figure 2:**
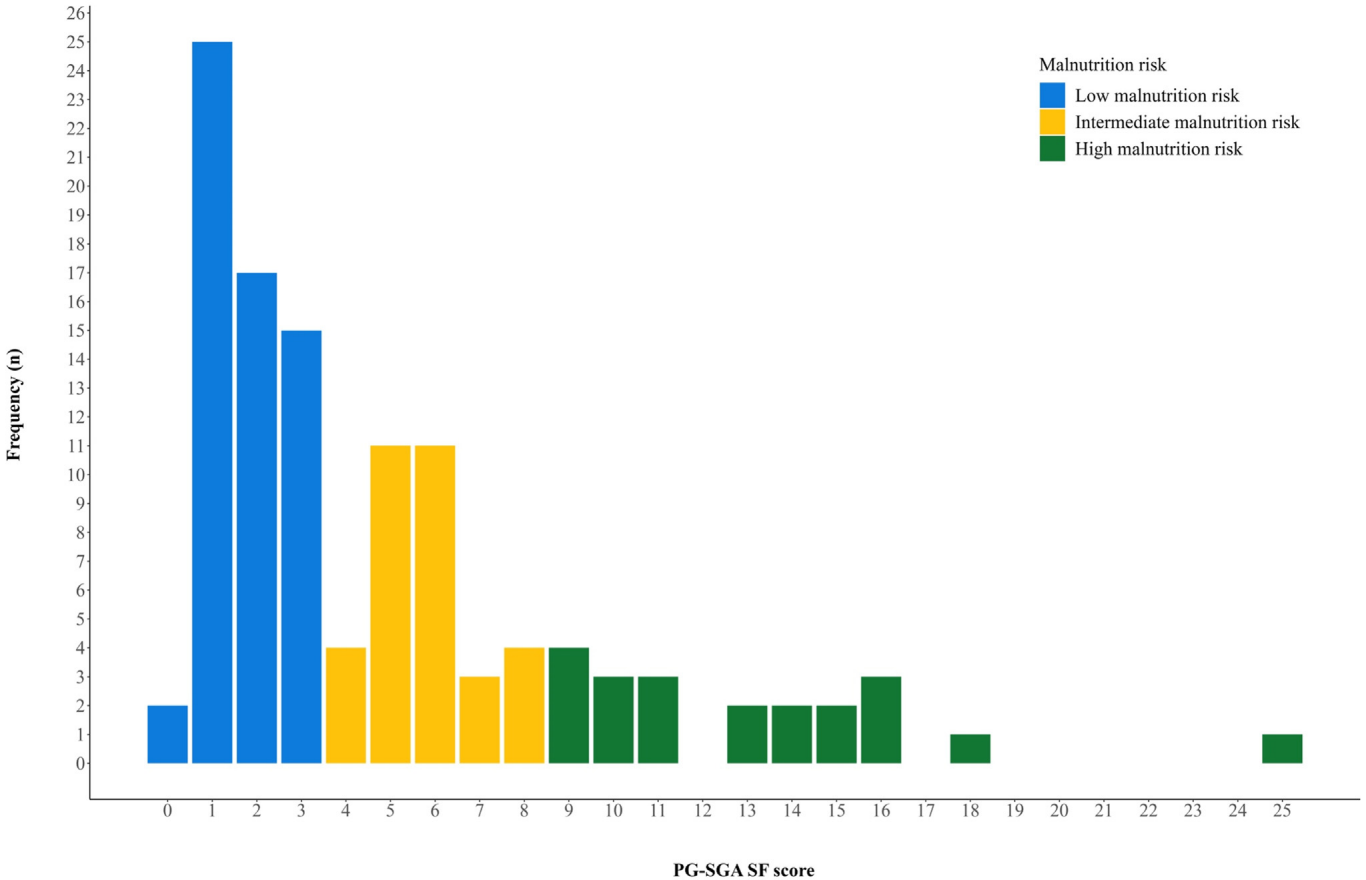
Total PG-SGA SF score and malnutrition risk categories. PG-SGA SF, Patient-Generated Subjective Global Assessment Short Form.

The median PG-SGA SF score for all patients was 3.0 (min 0.0, max 25.0; interquartile range: 2–6). Fifty-nine patients (52.2%) had low malnutrition risk, 33 (29.2%) had intermediate risk, and 21 (18.6%) had high risk (Fig. 2). The groups were well balanced for most characteristics, though the proportions with stage III disease, Eastern Cooperative Oncology Group PS 1 to 2, pleural fluid, weight loss 5% or higher three months before enrolment, and current smokers were higher in the high malnutrition risk group (Table 1). Supplementary Table 1 summarizes the most frequently reported nutrition impact symptoms (PG-SGA SF Box 3).

### Treatment Completion

One hundred four patients (92.0%) received all four chemotherapy courses and 109 patients (96.5%) completed TRT as planned. Between the three malnutrition risk categories, there were no significant differences in mean number of chemotherapy courses received (low malnutrition risk: 3.85, intermediate: 3.88, high: 3.90; *p* = 0.85), TRT completion (low: 96.6%, intermediate: 97.0%, high: 95.2%; *p* = 1.0) or proportions who received PCI (low: 78.0%, intermediate: 84.9%, high: 81.0%; *p* = 0.83).

Between patients with weight loss and without before enrolment, there were no significant differences in mean number of chemotherapy courses received (≥5%: 3.84, <5%: 3.86; *p* = 0.89), TRT completion (weight loss ≥5%: 96.0%, <5%: 96.1%; *p* = 1.0) or PCI (≥5%: 72.0%, <5%: 80.5%; *p* = 0.37).

### Toxicity

Fifty-two patients (88.1%) with low, 30 patients (90.9%) with intermediate, and 18 patients (85.7%) with a high risk of malnutrition experienced any grade 3 to 4 toxicity (*p* = 0.86) (Table 2). Between the three malnutrition risk categories, there were no significant differences in the proportions who experienced any grade 3 to 4 hematological, any grade 3 to 4 non-hematological, the most common grade 3 to 4 adverse events (anemia, neutropenia, thrombocytopenia, neutropenic infections, esophagitis) or pneumonitis, nor when analyzing TRT groups separately (Supplementary Table 2).

**Table 2:** Frequencies of the Most Common Grade 3 to 4 Toxicities and Pneumonitis in Each Malnutrition Risk Group

Toxicity Event	Low Malnutrition Risk (n = 59), n (%)	Intermediate Malnutrition Risk (n = 33), n (%)	High Malnutrition Risk (n = 21), n (%)	*p* value
	Grade 3–4	Grade 3–4	Grade 3–4	
Any toxicity	52 (88.1)	30 (90.9)	18 (85.7)	0.86
Any non-hematological	30 (50.9)	21 (63.6)	13 (61.9)	0.46
Any hematological	51 (86.4)	29 (87.9)	18 (85.7)	1.0
Anemia	9 (15.3)	6 (18.2)	7 (33.3)	0.22
Neutropenia	49 (83.1)	28 (84.9)	17 (81.0)	0.95
Thrombocytopenia	15 (25.4)	10 (30.3)	3 (14.3)	0.42
Neutropenic infection	20 (33.9)	11 (33.3)	9 (42.9)	0.73
Esophagitis	11 (18.6)	10 (30.3)	6 (28.6)	0.37
Pneumonitis	1 (1.7)	1 (3.0)	-	1.0

Twenty-three patients (92.0%) with weight loss before enrolment and 67 (87.0%) patients without weight loss experienced any grade 3 to 4 toxicity (*p* = 0.73). Comparing patients with and without weight loss, there were no significant differences in the proportions who experienced any grade 3 to 4 hematological toxicity (*p* = 0.51), any grade 3 to 4 non-hematological (*p* = 0.82), the most common grade 3 to 4 adverse events (neutropenia: *p* = 1.0; neutropenic infections: *p* = 0.81; thrombocytopenia: *p* = 1.0; esophagitis: *p* = 0.3;), or pneumonitis (*p* = 1.0) except for anemia (<5%: 9 patients [36.0%]; ≥5%: 11 patients [14.3%]; *p* = 0.039).

There were three treatment-related deaths, one in each malnutrition risk group (neutropenic fever, thrombocytopenic bleeding, and pneumonitis, in the low, intermediate, and high malnutrition risk groups, respectively).

Malnutrition risk was not a significant predictor for any grade 3 to 4 toxicity event in either univariable or multivariable analyses (Table 3). Weight loss of 5% or higher before enrolment was not a significant predictor for any grade 3 to 4 toxicity event in either univariable (odds ratio = 1.7, 95% CI: 0.4–11.7, *p* = 0.51) or multivariable analyses (odds ratio = 1.9, 95% CI: 0.4–13.8, *p* = 0.48).

**Table 3:** Multivariable Analyses for Progression-Free Survival, Overall Survival, and Any Toxicity CTCAE v.4.0 Grade 3 to 4

Analyzed Variable	Progression-Free Survival		Overall Survival		Any Toxicity Grade 3–4	
	Hazard Ratio (95% CI)	*p* value	Hazard Ratio (95% CI)	*p* value	Odds Ratio (95% CI)	*p* value
Malnutrition risk						
Low (n = 59)	1 (ref)	-	(1 ref)	-	1 (ref)	-
Intermediate (n = 33)	1.38 (0.83–2.30)	0.21	1.49 (0.90–2.46)	0.12	1.37 (0.32–7.28)	0.69
High (n = 21)	0.67 (0.34–1.33)	0.25	0.81 (0.42–1.58)	0.54	0.70 (0.14–4.07)	0.66
Age	1.00 (0.97–1.03)	0.97	1.02 (0.99–1.05)	0.27	1.01 (0.93–1.1)	0.89
Sex						
Male	1 (ref)	-	1 (ref)	-	1 (ref)	-
Female	0.76 (0.49–1.17)	0.22	0.82 (0.52–1.28)	0.38	2.16 (0.64–7.92)	0.22
Disease stage						
I–II	1 (ref)	-	1 (ref)	-	1 (ref)	-
III	2.22 (1.04–4.75)	0.041	2.16 (1.01–4.63)	0.047	2.70 (0.49–12.41)	0.21
Performance status						
0	1 (ref)	-	1 (ref)	-	1 (ref)	-
1	1.09 (0.68–1.75)	0.72	1.25 (0.77–2.04)	0.37	1.28 (0.32–5.23)	0.73
2	1.95 (0.93–4.07)	0.08	1.78 (0.87–3.64)	0.12	0.42 (0.08–2.61)	0.32
Treatment group						
45 Gy	1 (ref)	-	1 (ref)	-	1 (ref)	-
60 Gy	0.88 (0.56–1.38)	0.58	0.76 (0.48–1.20)	0.23	1.47 (0.44–5.12)	0.53

CI, confidence interval; CTCAE, Common Terminology Criteria for Adverse Events; ref, reference group.

### PFS and OS

**Figure 3:**
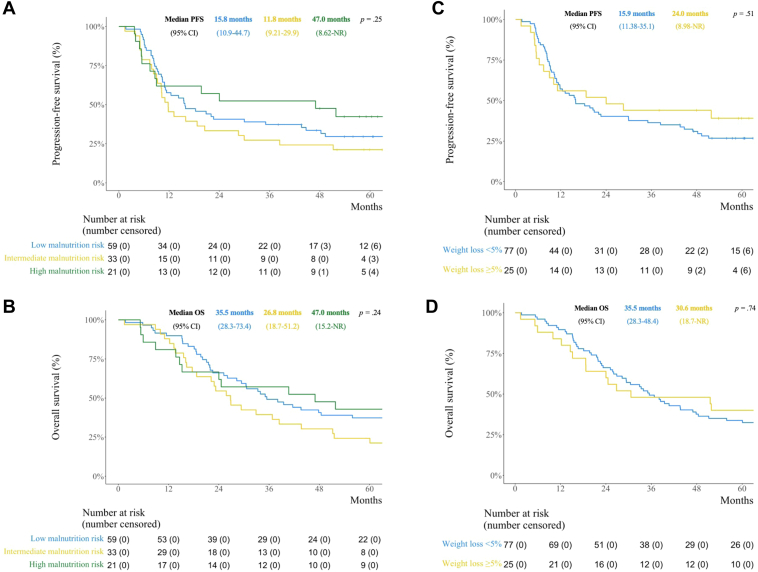
Kaplan-Meier survival analysis and log-rank test for and in included patients. (*A*) PFS for malnutrition risk categories. (*B*) OS for malnutrition risk categories. (*C*) PFS for weight loss categories. (*D*) OS for weight loss categories. CI, confidence interval; OS, overall survival; NR, not reached; PFS, progression-free survival.

There were no significant differences in median PFS between patients in the low (15.8 months, 95% CI: 10.9–44.7), intermediate (11.8 months, 95% CI: 9.2–29.9), or high (47.0 months, 95% CI: 8.62–not reached [NR]) malnutrition risk groups (*p* = 0.25) (Fig. 3*A*), nor when analyzing TRT-groups separately (Supplementary Fig. 3). Nor was malnutrition risk significantly associated with PFS in the multivariable analysis (Table 3). Total PG-SGA SF score was not significantly associated with PFS in either the univariable (hazard ratio [HR] = 0.98, 95% CI: 0.93–1.03) or the multivariable analysis (HR = 0.97, 95% CI: 0.92–1.02). The only significant predictor of PFS in the multivariable models was stage III disease (Table 3).

There were no significant differences in median OS between the low (35.5 months, 95% CI 28.3–73.4), intermediate (26.8 months, 95% CI: 18.7–51.2), or high malnutrition risk groups (47.0 months, 95% CI: 15.2–NR) (*p* = 0.24) (Fig. 3*B*). Nor was malnutrition risk significantly associated with OS in the multivariable analysis (Table 3). Total PG-SFA SF score was not a significant prognostic factor for OS neither in the univariable (HR = 1.0, 95% CI: 0.95–1.05) nor in the multivariable analysis (HR = 1.0, 95% CI: 0.93–1.04). The only significant predictor of OS in the multivariable models was stage III disease (Table 3).

There were no significant differences in PFS (median = 24.0 months, 95% CI: 8.98–NR, versus 15.9 months, 95% CI: 11.38–35.1, *p* = 0.51) (Fig. 3*C*) or OS (median = 30.6 months, 95% CI: 18.7–NR, versus 35.5 months, 95% CI: 28.3–48.4, *p* = 0.74) (Fig. 3*D*) between patients with and without weight loss three months before enrolment. Weight loss of 5% or higher three months before enrolment was not a significant predictor of either PFS or OS in the multivariable analyses. When analyzing TRT groups separately, there was significantly longer PFS among patients with pretreatment weight loss in the 60 Gy group. No other significant differences were revealed (Supplementary Fig. 3).

## Discussion

In our cohort of patients with LS SCLC receiving concurrent CRT, we found that 29.2% of patients had intermediate and 18.6% high risk of malnutrition at baseline according to patient reports on the PG-SGA SF. Interestingly, there were no significant differences in treatment completion rates, treatment toxicity, PFS, or OS between the malnutrition risk groups. Furthermore, weight loss of 5% or more in the last three months before CRT was not associated with more treatment toxicity or inferior PFS or OS.

Although we used patient reports whereas other studies have used nutrition-inflammation prognostic indices to assess nutritional status, such as the Cachexia Index,^30^ Controlling Nutritional Status,^31^ and Prognostic Nutritional Index,^32^ the prevalence of malnutrition in our cohort is similar to what is found in other cohorts of LS SCLC: poor nutritional status in up to 57.4%,^33^ and moderately and severely poor nutritional status in up to 32.3% and 18.3% of patients,^34^ respectively.

The proportion of patients with weight loss in our cohort is in the upper range of what has previously been reported among LS SCLC patients receiving CRT. The Intergroup 0096 and CALGB studies reported more than 5% weight loss six months before enrolment in 9.1% and 15.8% of patients, respectively.^35^^,^^36^ In other studies, the incidence of weight loss in LS SCLC patients before CRT commenced ranged from 10.7% to 24.4%, though the percentage of weight loss differed, possibly because the period of weight loss was not always reported.^37^, ^38^, ^39^ The possible higher proportion of patients with weight loss in our trial might be explained by fewer restrictions with respect to eligibility criteria for the trial, slightly higher median age, and a more liberal definition of “limited stage” than in other studies of LS SCLC,^35^^,^^36^^,^^38^^,^^40^ and that our proportion of patients with a PS of 2 was higher.^40^

Guidelines and reviews state that weight loss and malnutrition before cancer treatment are associated with an increased risk of severe toxicity.^10^^,^^41^^,^^42^ Nevertheless, in line with our results, previous studies of SCLC have not revealed such associations with chemotherapy toxicity.^35^^,^^43^^,^^44^

In contrast to our findings, previous studies conclude that poor nutritional status and pre-treatment weight loss are associated with shorter PFS and OS in SCLC.^11^^,^^38^^,^^39^^,^^43^ Nevertheless, these studies have used other methods for assessing nutritional status, various cut-off values, and different periods of interest for malnutrition and weight loss. The nutrition-inflammation prognostic indices used to assess malnutrition are on the basis of lymphocyte count and cholesterol and albumin values only, for which clinical cutoff values are not established and associations with patient reports are poorly defined.^45^ Furthermore, some of these blood values might be influenced by other factors; e.g. hypoalbuminemia may be caused by inflammation.^45^^,^^46^ Moreover, these studies included patients with both LS and ES SCLC and patients underwent various therapies. Another difference is that we analyzed participants in a randomized trial, who often have better health than many patients seen in routine clinical practice.

The main limitation of our study is the sample size, and this might explain why we, in contrast to previous studies of patients with cancer,^24^ did not find any associations between PG-SGA SF malnutrition risk and safety or efficacy. Nevertheless, the biology of SCLC is different from most other solid tumors, and the impact of nutritional status and outcomes in SCLC is less established than for other types of cancers such as NSCLC, possibly because the response to both chemo- and radiotherapy is more rapid and profound than for most other cancers. We did not record why some patients did not complete the baseline PG-SGA SF or did not report weight loss at the time of inclusion, but baseline characteristics between these patients and the present study cohort were comparable (Table 1), suggesting that non-completion was most probably owing to suboptimal routines at some sites.

The PG-SGA has high construct and content validity and is widely acknowledged in clinical research and practice as the reference method for evaluating nutritional status in patients with catabolic conditions generally, and cancer specifically. The PG-SGA SF does not provide a full assessment of nutritional status but was considered appropriate for this exploratory sub-study of our trial. In a previous study, we did not find any associations between body composition assessed from computed tomography images and treatment outcomes,^47^ and we were not aware of any other data suggesting a strong clinical impact of a more extensive assessment of nutritional status in this setting.

Similar to previous studies, we were not able to accurately reveal the causality of patients' weight loss or poor nutritional status. It is possible that the impacts of nutritional status and weight loss vary depending on whether these are due to comorbidities or the cancer itself.^48^ Furthermore, side effects from chemoradiotherapy might impact nutritional status, especially among those who experience severe radiation esophagitis. Unfortunately, our study was not designed to assess such potential impact, and data on interventions such as referral to a dietician or active nutritional support before or during study treatment are not available in either our study or previous studies.

Despite this study's limitations, it is to our knowledge the most comprehensive study of the potential clinical role of pre-treatment nutritional challenges among patients with LS SCLC receiving standard concurrent CRT using a validated tool for assessing nutritional status. Our findings suggest that also LS SCLC patients with malnutrition tolerate CRT and respond well and rapidly to such therapy, and supposably it is possible to reverse catabolic processes among patients who achieve good tumor control.

## Conclusion

In conclusion, we found that almost half of our study population had intermediate or high malnutrition risk, and approximately one-fifth had a weight loss of more than 5% at baseline. Nevertheless, these patients did not experience more toxicity, shorter PFS, or inferior OS, suggesting that also LS SCLC patients with suboptimal nutritional status should be offered standard CRT.

## CRediT Authorship Contribution Statement

**Evgenia Taranova:** Writing - original draft, Writing - review & editing, Formal analysis, Data curation**,** Visualization.

**Marianne Aanerud:** Investigation, Resources, Writing - original draft, Writing - review & editing, Supervision.

**Tarje Onsøien Halvorsen:** Methodology, Validation, Investigation, Resources, Data curation, Writing - review & editing, Supervision.

**Kristin Toftaker Killingberg:** Methodology, Validation, Investigation, Resources, Data curation, Writing - review & editing, Supervision.

**Marit Slaaen:** Writing - reviewing & editing.

**Bjørn Henning Grønberg:** Conceptualization, Methodology, Investigation, Writing - original draft, Writing - review & editing, Supervision, Project administration, Funding acquisition.

## Disclosure

The authors declare no conflict of interest.

## References

[1] Rudin C.M., Brambilla E., Faivre-Finn C., Sage J. (2021). Small-cell lung cancer. Nat Rev Dis Primers.

[2] Rudin C.M., Ismaila N., Hann C.L. (2015). Treatment of small-cell lung cancer: American Society of Clinical Oncology endorsement of the American College of Chest Physicians guideline. J Clin Oncol.

[3] Damhuis R., Widder J., Senan S. (2018). Population-based results of chemoradiotherapy for limited stage small cell lung cancer in the Netherlands. Clin Oncol (R Coll Radiol).

[4] Levy A., Hendriks L.E.L., Le Péchoux C. (2019). Current management of limited-stage SCLC and CONVERT trial impact: results of the EORTC Lung Cancer Group survey. Lung Cancer.

[5] Dingemans A.C., Früh M., Ardizzoni A. (2021). Small-cell lung cancer: ESMO clinical practice guidelines for diagnosis, treatment and follow-up. Ann Oncol.

[6] Stokes M., Berfeld N., Gayle A., Descoteaux A., Rohrmoser O., Franks A. (2022). A systematic literature review of real-world treatment outcomes of small cell lung cancer. Medicine (Baltimore).

[7] Schreiber D., Wong A.T., Schwartz D., Rineer J. (2015). Utilization of hyperfractionated radiation in small-cell lung cancer and its impact on survival. J Thorac Oncol.

[8] Glatzer M., Faivre-Finn C., De Ruysscher D. (2020). Once daily versus twice-daily radiotherapy in the management of limited disease small cell lung cancer - Decision criteria in routine practise. Radiother Oncol.

[9] Capra S., Ferguson M., Ried K. (2001). Cancer: impact of nutrition intervention outcome--nutrition issues for patients. Nutrition.

[10] Arends J., Bachmann P., Baracos V. (2017). ESPEN guidelines on nutrition in cancer patients. Clin Nutr.

[11] Dewys W.D., Begg C., Lavin P.T. (1980). Prognostic effect of weight loss prior to chemotherapy in cancer patients. Eastern Cooperative Oncology Group. Am J Med.

[12] Rivadeneira D.E., Evoy D., Fahey T.J., Lieberman M.D., Daly J.M. (1998). Nutritional support of the cancer patient. CA Cancer J Clin.

[13] Bradley J., Movsas B. (2004). Radiation esophagitis: predictive factors and preventive strategies. Semin Radiat Oncol.

[14] Baker S., Fairchild A. (2016). Radiation-induced esophagitis in lung cancer. Lung Cancer (Auckl).

[15] Bradley J.D., Paulus R., Komaki R. (2015). Standard-dose versus high-dose conformal radiotherapy with concurrent and consolidation carboplatin plus paclitaxel with or without cetuximab for patients with stage IIIA or IIIB non-small-cell lung cancer (RTOG 0617): a randomised, two-by-two factorial phase 3 study. Lancet Oncol.

[16] Demedts I.K., Vermaelen K.Y., van Meerbeeck J.P. (2010). Treatment of extensive-stage small cell lung carcinoma: current status and future prospects. Eur Respir J.

[17] Ottery F.D. (1996). Definition of standardized nutritional assessment and interventional pathways in oncology. Nutrition.

[18] Azevedo M.D., de Pinho N.B., de Carvalho Padilha P., de Oliveira L.C., Peres W.A.F. (2024). Clinical usefulness of the patient-generated subjective global assessment short form© for nutritional screening in patients with head and neck cancer: a multicentric study. Ecancermedicalscience.

[19] Dewansingh P., Euwes M., Krijnen W.P., Strijbos J.H., van der Schans C.P., Jager-Wittenaar H. (2021). Patient-generated subjective global assessment short form better predicts length of stay than short nutritional assessment questionnaire. Nutrition.

[20] Abbott J., Teleni L., McKavanagh D., Watson J., McCarthy A.L., Isenring E. (2016). Patient-Generated Subjective Global Assessment Short Form (PG-SGA SF) is a valid screening tool in chemotherapy outpatients. Support Care Cancer.

[21] Stoyanoff L., Leung E., Robinson J. (2009). Validation of the abridged patient-generated subjective global assessment as a screening tool for malnutrition in an outpatient oncology setting. J Am Diet Assoc.

[22] Vigano A.L., di Tomasso J., Kilgour R.D. (2014). The abridged patient-generated subjective global assessment is a useful tool for early detection and characterization of cancer cachexia. J Acad Nutr Diet.

[23] Jager-Wittenaar H., Ottery F.D. (2017). Assessing nutritional status in cancer: role of the patient-generated subjective global assessment. Curr Opin Clin Nutr Metab Care.

[24] Zhang J., Xu W., Zhang H., Fan Y. (2024). Association between risk of malnutrition defined by patient-generated subjective global assessment and adverse outcomes in patients with cancer: a systematic review and meta-analysis. Public Health Nutr.

[25] Grønberg B.H., Killingberg K.T., Fløtten Ø. (2021). High-dose versus standard-dose twice-daily thoracic radiotherapy for patients with limited stage small-cell lung cancer: an open-label, randomised, phase 2 trial. Lancet Oncol.

[26] Sealy M.J., Nijholt W., Stuiver M.M. (2016). Content validity across methods of malnutrition assessment in patients with cancer is limited. J Clin Epidemiol.

[27] Banning L.B.D., ter Beek L., El Moumni M. (2020). Vascular surgery patients at risk for malnutrition are at an increased risk of developing postoperative complications. Ann Vasc Surg.

[28] Jager-Wittenaar H., de Bats H.F., Welink-Lamberts B.J. (2020). Self-completion of the patient-generated subjective global assessment short form is feasible and is associated with increased awareness on malnutrition risk in patients with head and neck cancer. Nutr Clin Pract.

[29] Beek L.T., Banning L.B.D., Visser L. (2018). Risk for malnutrition in patients prior to vascular surgery. Am J Surg.

[30] Go S.I., Park M.J., Lee G.W. (2021). Clinical significance of the cachexia index in patients with small cell lung cancer. BMC Cancer.

[31] Ignacio de Ulíbarri J., González-Madroño A., de Villar N.G. (2005). CONUT: a tool for controlling nutritional status. First validation in a hospital population. Nutr Hosp.

[32] Onodera T., Goseki N., Kosaki G. (1984). [Prognostic nutritional index in gastrointestinal surgery of malnourished cancer patients]. Nihon Geka Gakkai Zasshi.

[33] Hong S., Zhou T., Fang W. (2015). The prognostic nutritional index (PNI) predicts overall survival of small-cell lung cancer patients. Tumour Biol.

[34] Li L., Wang Y., Yang P. (2021). Correlation of the controlling nutritional status score and the prognostic nutritional index with the prognosis of patients treated with radiotherapy for small-cell lung cancer. Ann Palliat Med.

[35] Turrisi A.T., Kim K., Blum R. (1999). Twice-daily compared with once-daily thoracic radiotherapy in limited small-cell lung cancer treated concurrently with cisplatin and etoposide. N Engl J Med.

[36] Bogart J., Wang X., Masters G. (2023). High-dose once-daily thoracic radiotherapy in limited-stage small-cell lung cancer: CALGB 30610 (alliance)/RTOG 0538. J Clin Oncol.

[37] Gregor A., Drings P., Burghouts J. (1997). Randomized trial of alternating versus sequential radiotherapy/chemotherapy in limited-disease patients with small-cell lung cancer: a European Organization for Research and Treatment of Cancer Lung Cancer Cooperative Group Study. J Clin Oncol.

[38] Xia B., Chen G.Y., Cai X.W. (2011). The effect of bioequivalent radiation dose on survival of patients with limited-stage small-cell lung cancer. Radiat Oncol.

[39] Bremnes R.M., Sundstrom S., Aasebø U. (2003). The value of prognostic factors in small cell lung cancer: results from a randomised multicenter study with minimum 5 year follow-up. Lung Cancer.

[40] Faivre-Finn C., Snee M., Ashcroft L. (2017). Concurrent once-daily versus twice-daily chemoradiotherapy in patients with limited-stage small-cell lung cancer (CONVERT): an open-label, phase 3, randomised, superiority trial. Lancet Oncol.

[41] Van Cutsem E., Arends J. (2005). The causes and consequences of cancer-associated malnutrition. Eur J Oncol Nurs.

[42] Langer C.J., Hoffman J.P., Ottery F.D. (2001). Clinical Significance of weight loss in cancer patients: rationale for the use of anabolic agents in the treatment of cancer-related cachexia. Nutrition.

[43] Ross P.J., Ashley S., Norton A. (2004). Do patients with weight loss have a worse outcome when undergoing chemotherapy for lung cancers?. Br J Cancer.

[44] Jereczek-Fossa B., Jassem J., Karnicka-Młodkowska H. (1998). Does chemotherapy-induced leukopenia predict a response in small-cell lung cancer?. J Cancer Res Clin Oncol.

[45] Bharadwaj S., Ginoya S., Tandon P. (2016). Malnutrition: laboratory markers vs nutritional assessment. Gastroenterol Rep (Oxf).

[46] Soeters P.B., Wolfe R.R., Shenkin A. (2019). Hypoalbuminemia: pathogenesis and clinical significance. JPEN J Parenter Enteral Nutr.

[47] Halvorsen T.O., Valan C.D., Slaaen M., Grønberg B.H. (2020). Associations between muscle measures, survival, and toxicity in patients with limited stage small cell lung cancer. J Cachexia Sarcopenia Muscle.

[48] Alexander M., Evans S.M., Stirling R.G. (2016). The influence of comorbidity and the simplified comorbidity score on overall survival in non-small cell lung cancer-a prospective cohort study. J Thorac Oncol.

