# Supplementary material for: Associations Between Patient-Reported Nutritional Status, Toxicity, and Survival in Limited-Stage SCLC
Source: JTO Clin Res Rep. 2024 Nov 12;6(1):100764. doi: 10.1016/j.jtocrr.2024.100764 (PMC11719838; doi:10.1016/j.jtocrr.2024.100764)
Supplement: Supplemental Material [file mmc1.pdf]

Title: “Supplementary Figure 1: Patient-Generated Subjective Global Assessment Short Form (PG-SGA SF) ©FD Ottery 2005, 2006, 2015, 2020 v4.3.20”

Scored Patient-Generated Subjective Global Assessment (PG-SGA)

History: Boxes 1 - 4 are designed to be completed by the patient.
[Boxes 1-4 are referred to as the PG-SGA Short Form (SF)]

Patient Identification Information

1. Weight *(See Worksheet 1)*

In summary of my current and recent weight:

I currently weigh about _____kg
I am about _____ cm tall

One month ago I weighed about _____ kg
Six months ago I weighed about _____ kg

During the past two weeks my weight has:

☐ decreased (1) ☐ not changed (0) ☐ increased (0)

Box 1 ☐

2. Food intake: As compared to my normal intake, I would rate my food intake during the past month as

- ☐ unchanged (0)
- ☐ more than usual (0)
- ☐ less than usual (1)

I am now taking

- ☐ *normal food* but less than normal amount (1)
- ☐ little solid food (2)
- ☐ only liquids (3)
- ☐ only nutritional supplements (3)
- ☐ very little of anything (4)
- ☐ only tube feedings or only nutrition by vein (0)

Box 2 ☐

3. Symptoms: I have had the following problems that have kept me from eating enough during the past two weeks (check all that apply)

- ☐ no problems eating (0)
- ☐ no appetite, just did not feel like eating (3)
- ☐ vomiting (3)
- ☐ nausea (1)
- ☐ diarrhea (3)
- ☐ constipation (1)
- ☐ dry mouth (1)
- ☐ mouth sores (2)
- ☐ smells bother me (1)
- ☐ things taste funny or have no taste (1)
- ☐ feel full quickly (1)
- ☐ problems swallowing (2)
- ☐ fatigue (1)
- ☐ pain; where? (3) ________________
- ☐ other (1)** ____________________

**Examples: depression, money, or dental problems

Box 3 ☐

4. Activities and Function:
Over the past month, I would generally rate my activity as:

- ☐ normal with no limitations (0)
- ☐ not my normal self, but able to be up and about with fairly normal activities (1)
- ☐ not feeling up to most things, but in bed or chair less than half the day (2)
- ☐ able to do little activity and spend most of the day in bed or chair (3)
- ☐ pretty much bed ridden, rarely out of bed (3)

Box 4 ☐

The remainder of this form is to be completed by your doctor, nurse, dietitian, or therapist. Thank you.

Additive Score of Boxes 1-4 ☐ **A**

©FD Ottery 2005, 2006, 2015, 2020 v4.3.20
email: faithotterymdphd@gmail.com or info@pt-global.org

Title: «Supplementary Table 1: Frequencies of symptoms influencing nutritional status listed in the Patient-Generated Subjective Global Assessment Short Form (Box 3), split for malnutrition risk groups»
Description: -

	Low malnutrition risk (n=59)		Intermediate malnutrition risk (n=33)		High malnutrition risk (n=21)		Total (n=113)	
**«No problems eating»**	56	94.9%	19	57.6%	4	19.0%	79	69.9%
**«No appetite, just did not feel like eating»**	0	0%	3	9.1%	15	71.4%	18	15.9%
**«Nausea»**	1	1.7%	7	21.2%	11	52.4%	19	16.8%
**«Vomiting»**	0	0%	1	3.0%	5	23.8%	6	5.3%
**«Diarrhea»**	0	0%	1	3.0%	4	19.0%	5	4.4%
**«Constipation»**	1	1.7%	5	15.2%	6	28.6%	12	10.6%
**«Dry mouth»**	2	3.4%	7	21.2%	5	23.8%	14	12.4%
**«Mouth sores»**	0	0%	0	0%	4	19.0%	4	3.5%
**«Smells bother me»**	0	0%	1	3.0%	9	42.9%	10	8.8%
**«Things taste funny or have no taste»**	0	0%	4	12.1%	10	47.6%	14	12.4%
**«Feel full quickly»**	3	5.1%	9	27.3%	11	52.4%	23	20.4%
**«Problems swallowing»**	1	1.7%	3	9.1%	5	23.8%	9	8.0%
**«Fatigue»**	1	1.7%	2	6.1%	2	9.5%	5	4.4%
**«Pain»**	1	1.7%	2	6.1%	5	23.8%	8	7.1%
**«Other»**	1	1.7%	1	3.0%	2	9.5%	4	3.5%

Title: "Supplementary Table 2: Risk of severe toxicity across malnutrition risk groups within each treatment arm"

		Any toxicity grade 3-4	
		**Odds ratio (95% CI)**	**p value**
**High-dose 60 Gy/40 fractions (n=60)**	**Malnutrition risk**		
	**Low** (n=13)	1 (ref)	-
	**Intermediate** (n=17)	1.14 (0.10-25.73)	0.92
	**High** (n=30)	0.24 (0.03-1.63)	0.15
	**Weight loss**		
	**< 5%**	1 (ref)	-
	**≥ 5%**	1 (0.13-20.7)	1
**Low-dose 45 Gy/30 fractions(n=53)**	**Malnutrition risk**		
	**Low** (n=8)	1 (ref)	-
	**Intermediate** (n=16)	1.46 (0.27-11.13)	0.68
	**High** (n=29)	$24 \cdot 10^6$ (0.00-NA)	0.99
	**Weight loss**		
	**< 5%**	1 (ref)	-
	**≥ 5%**	2.89 (0.43-57.55)	0.35

Title: **“**Supplementary Figure 2: Progression free (PFS) and overall survival (OS) according to malnutrition risk groups within each trial group. A) PFS in the 60 Gy group, B) PFS in the 45 Gy group, C) OS in the 60 Gy group, and D) OS in the 45 Gy group”

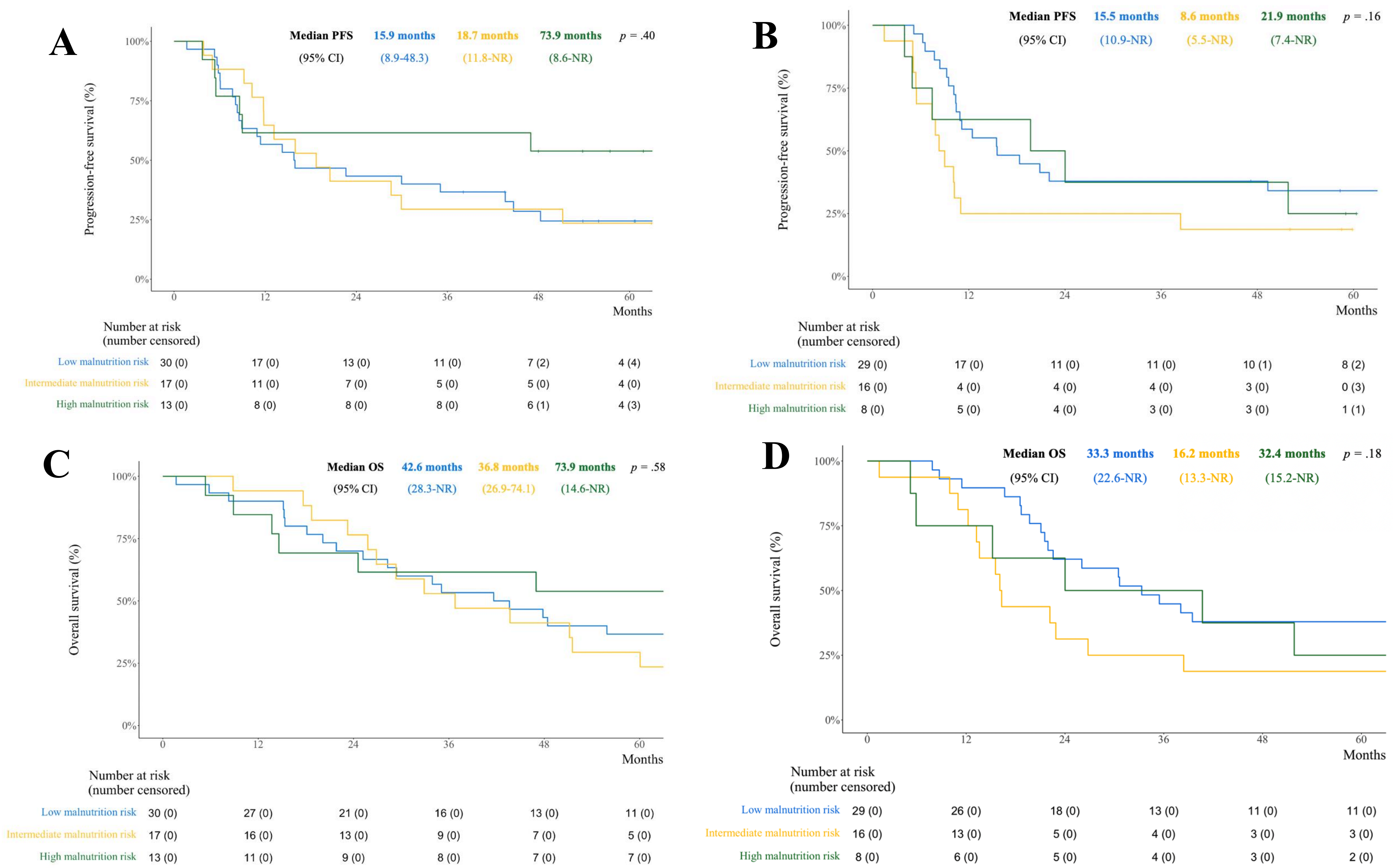
A
Median PFS 15.9 months 18.7 months 73.9 months p = .40
(95% CI) (8.9-48.3) (11.8-NR) (8.6-NR)
Progression-free survival (%)
Months
Number at risk
(number censored)
Low malnutrition risk 30 (0) 17 (0) 13 (0) 11 (0) 7 (2) 4 (4)
Intermediate malnutrition risk 17 (0) 11 (0) 7 (0) 5 (0) 5 (0) 4 (0)
High malnutrition risk 13 (0) 8 (0) 8 (0) 8 (0) 6 (1) 4 (3)
B
Median PFS 15.5 months 8.6 months 21.9 months p = .16
(95% CI) (10.9-NR) (5.5-NR) (7.4-NR)
Progression-free survival (%)
Months
Number at risk
(number censored)
Low malnutrition risk 29 (0) 17 (0) 11 (0) 11 (0) 10 (1) 8 (2)
Intermediate malnutrition risk 16 (0) 4 (0) 4 (0) 4 (0) 3 (0) 0 (3)
High malnutrition risk 8 (0) 5 (0) 4 (0) 3 (0) 3 (0) 1 (1)
C
Median OS 42.6 months 36.8 months 73.9 months p = .58
(95% CI) (28.3-NR) (26.9-74.1) (14.6-NR)
Overall survival (%)
Months
Number at risk
(number censored)
Low malnutrition risk 30 (0) 27 (0) 21 (0) 16 (0) 13 (0) 11 (0)
Intermediate malnutrition risk 17 (0) 16 (0) 13 (0) 9 (0) 7 (0) 5 (0)
High malnutrition risk 13 (0) 11 (0) 9 (0) 8 (0) 7 (0) 7 (0)
D
Median OS 33.3 months 16.2 months 32.4 months p = .18
(95% CI) (22.6-NR) (13.3-NR) (15.2-NR)
Overall survival (%)
Months
Number at risk
(number censored)
Low malnutrition risk 29 (0) 26 (0) 18 (0) 13 (0) 11 (0) 11 (0)
Intermediate malnutrition risk 16 (0) 13 (0) 5 (0) 4 (0) 3 (0) 3 (0)
High malnutrition risk 8 (0) 6 (0) 5 (0) 4 (0) 3 (0) 2 (0)

Title: “Supplementary Figure 3: Progression free (PFS) and overall survival (OS) according to weight loss within each trial group. A) PFS in the 60 Gy group, B) PFS in the 45 Gy group, C) OS in the 60 Gy group, and D) OS in the 45 Gy group”

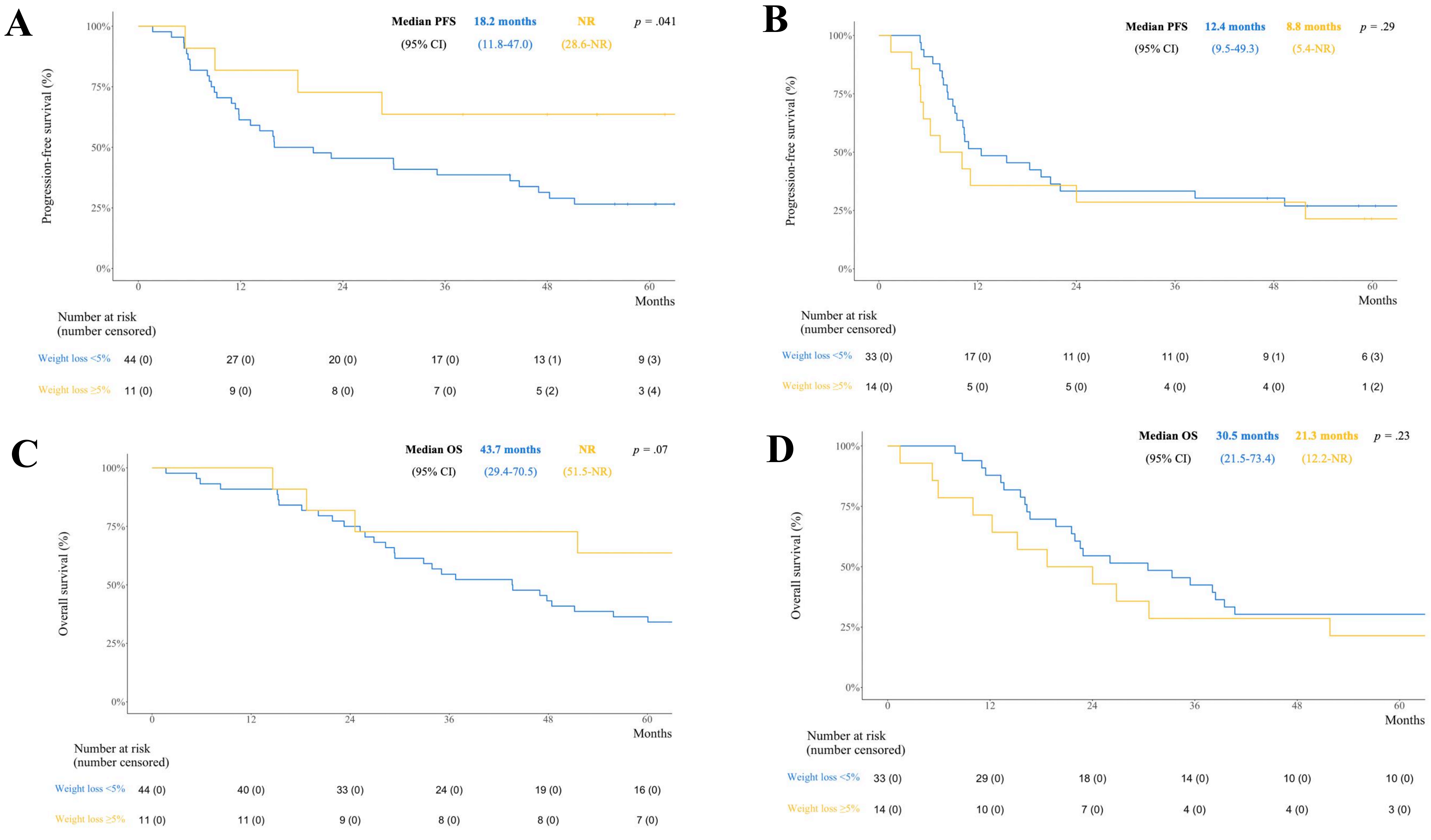
A
Median PFS
18.2 months
NR
p = .041
(95% CI)
(11.8-47.0)
(28.6-NR)
Progression-free survival (%)
100%
75%
50%
25%
0%
0
12
24
36
48
60
Months
Number at risk
(number censored)
Weight loss <5% 44 (0) 27 (0) 20 (0) 17 (0) 13 (1) 9 (3)
Weight loss ≥5% 11 (0) 9 (0) 8 (0) 7 (0) 5 (2) 3 (4)
B
Median PFS
12.4 months
8.8 months
p = .29
(95% CI)
(9.5-49.3)
(5.4-NR)
Progression-free survival (%)
Months
Number at risk
(number censored)
Weight loss <5% 33 (0) 17 (0) 11 (0) 11 (0) 9 (1) 6 (3)
Weight loss ≥5% 14 (0) 5 (0) 5 (0) 4 (0) 4 (0) 1 (2)
C
Median OS
43.7 months
NR
p = .07
(95% CI)
(29.4-70.5)
(51.5-NR)
Overall survival (%)
Months
Number at risk
(number censored)
Weight loss <5% 44 (0) 40 (0) 33 (0) 24 (0) 19 (0) 16 (0)
Weight loss ≥5% 11 (0) 11 (0) 9 (0) 8 (0) 8 (0) 7 (0)
D
Median OS
30.5 months
21.3 months
p = .23
(95% CI)
(21.5-73.4)
(12.2-NR)
Overall survival (%)
Months
Number at risk
(number censored)
Weight loss <5% 33 (0) 29 (0) 18 (0) 14 (0) 10 (0) 10 (0)
Weight loss ≥5% 14 (0) 10 (0) 7 (0) 4 (0) 4 (0) 3 (0)
